# Non-Toxin-Based *Clostridioides difficile* Vaccination Approaches

**DOI:** 10.3390/pathogens12020235

**Published:** 2023-02-02

**Authors:** Agnieszka Razim, Sabina Górska, Andrzej Gamian

**Affiliations:** Hirszfeld Institute of Immunology and Experimental Therapy, Polish Academy of Sciences, 53-114 Wrocław, Poland

**Keywords:** *Clostridioides difficile* infection, vaccine, protein, polysaccharide, epitope, spore, mucosal immunity

## Abstract

*Clostridioides difficile* (CD) is a Gram-positive, anaerobic bacterium that infects mainly hospitalized and elderly people who have been treated with long-term antibiotic therapy leading to dysbiosis. The deteriorating demographic structure and the increase in the number of antibiotics used indicate that the problem of CD infections (CDI) will continue to increase. Thus far, there is no vaccine against CD on the market. Unfortunately, clinical trials conducted using the CD toxin-based antigens did not show sufficiently high efficacy, because they did not prevent colonization and transmission between patients. It seems that the vaccine should also include antigens found in the bacterium itself or its spores in order not only to fight the effects of toxins but also to prevent the colonization of the patient. This literature review summarizes the latest advances in research into vaccine antigens that do not contain CD toxins.

## 1. Introduction

*Clostridioides difficile* (CD) is an anaerobic Gram-positive opportunistic bacterium and a resident of gut microbiota in approximately 3% of older children and adults. Asymptomatic carriage is much more common in hospitalized patients and in healthcare professionals, with a CD incidence of 10–30% [1]. The bacterium becomes dangerous to the organism when dysbiosis develops, i.e., when the quantitative and qualitative composition of the intestinal bacterial microflora is disturbed [2]. Dysbiosis leading to the development of CDI (*Clostridioides difficile* infection) is usually caused by antibiotic treatment with clindamycin, cephalosporins, penicillin, or fluoroquinolones [3]. CD, which is resistant to these antibiotics, massively multiplies in the intestines, and then starts the production of toxins: TcdA and TcdB. Next, toxins damage the cytoskeleton of the gut cells and intercellular connections, which leads to the destruction of the intestinal epithelium and the accumulation of fluids, resulting in massive diarrhea [4]. Additionally, the bacteria produce vast amounts of spores that can survive for long periods on hospital surfaces such as bed backs and tabletops. Symptoms of CDI range from mild diarrhea and abdominal pain to *pseudomembranous colitis* and, in the most severe case, may lead to distention of the colon, perforation of the intestine, and even death [5]. The observations made by clinicians show that the risk group is constantly growing, and the disease increasingly affects patients under 60 years of age and without a history of hospitalization. The most available form of fighting the pathogen is antibiotic therapy; however, the bacteria show increasing resistance to the drugs used [6,7]. Other methods include probiotic therapy, the usage of monoclonal antibodies, and fecal transplant therapy. 

**Figure 1:**
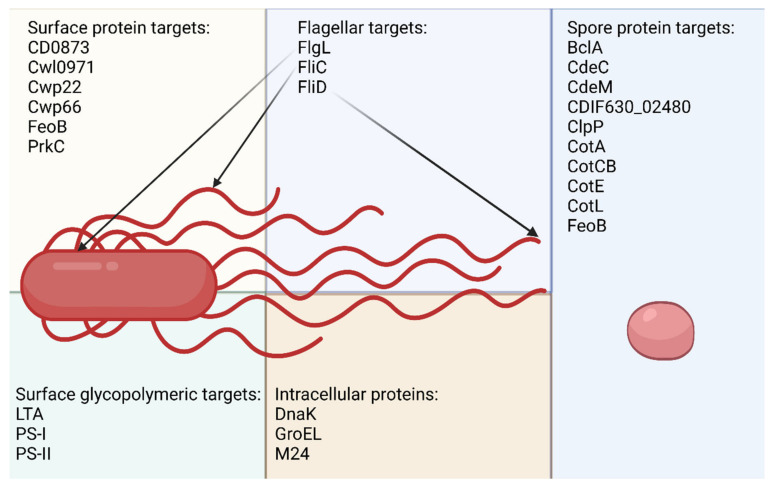
CD vaccine candidate antigens that were characterized in recent research papers. Created with BioRender.com.

Clinical trials are ongoing with three CD toxoid-based vaccines. Pfizer tested its CD vaccine candidate (PF-06425090) in phase III of clinical studies (NCT03090191). They have shown that their vaccine shortened the disease and lowered its severity but it did not prevent the initial infection. In addition, Sanofi-Pasteur was developing a vaccine (CDIFFENSE™) but discontinued the works after phase III of clinical trials. The third vaccine that is still in clinical trials is VLA84 (Valneva, Austria, NCT01296386, NCT02316470), consisting of a protein composed of truncated TcdA and TcdB amino acid sequences. However, the phase III clinical trial was put on hold. The Pfizer study proved what was already assumed by the scientists, that a vaccine containing only toxoids will not prevent transmission of bacteria between patients and will not eliminate infection at an early stage [8]. It becomes clear that potential vaccines should also include other antigens, such as surface proteins or polysaccharides, the immunization with which would respond earlier in the infection without leading to massive colonization and the production of toxins that destroy the patient’s gut. This minireview article covers the latest advances in the discovery of non-toxin-based CD vaccine antigens that were made over the past couple of years (Figure 1).

## 2. Targeting CD Protein Surface Components

Protein-based vaccines are gaining more and more interest as they have a much better safety profile than whole-microorganism vaccines. Moreover, their production cost is competitive with RNA vaccines and they do not require ultra-cold refrigeration. There are already antibacterial protein-based vaccines on the market (against pertussis, tetanus, or diphtheria). Others are in clinical studies such as M72, a recombinant fusion protein against tuberculosis consisting of two proteins: putative evasion factor (membrane-associated protein) and putative serine protease [9]. It seems that including surface-displayed proteins in vaccine preparations is a reasonable approach because they are readily available, immunogenic, and very often are virulence factors responsible for interaction with the host [10]. As CD exists both as a vegetative cell and spore, both of their surfaces are in the scope of new vaccine antigens searches. 

### 2.1. CD Spore Surface Proteins as Vaccine Candidates

Targeting proteins present on the surface of spores seems to be a rational approach as it could eliminate the threat at a very early stage, before the bacterium begins to produce destructive toxins. Spores and vegetative cells share 80% of proteins, which suggests a moderate proteomic changeover [11]; however, especially for the spore, most work is performed on the spore-specific proteins. There are 184 proteins in the CD exosporium layer; however, only a small group of them is immunogenic [12]. 

The BclA family of collagen-like glycoproteins cover the surface of CD spores (exosporium). BclaA2 and BclaA3 form hair-like structures on the spore surface of a hypervirulent CD strain R20291, which are easily accessible and interact with epithelial cells [13]. Therefore, this protein family was proposed as a suitable vaccine antigen by several groups. A 131-amino-acid-long fragment of BclA2 named BclA2_CDT_ was exposed on the surface of *Bacillus subtilis* spores to improve vaccine stability and delivery and administered intranasally to mice. The adsorbed BclA2_CDT_ as well as its free form induced a similar, specific humoral response. However, it was not enough to have a protective effect [14]. Another group used a recombined BclA3 peptide and the corresponding glycopeptide conjugated to the KLH (keyhole limpet hemocyanin) carrier protein and administered it intranasally to mice. Again, although specific antibodies were raised to both antigens, immunization did not provide any protection against acute or recurrent disease [15]. One of the reasons might be that spore coat proteins cover the exosporium proteins, therefore preventing antibody binding [16]. The conjugate of exosporium protein CdeM fused to the carboxy-terminus of the B subunit of the *Escherichia coli* heat-labile enterotoxin (LTB) was used to design a plant-based vaccine against CD. Transformed tobacco plants efficiently produced the LTB-CdeM antigen that was orally immunogenic [17]. CdeM was already shown to induce a high level of protection in CDI animal models [18]. 

CotE is a bi-functional spore coat protein that carries an N-terminal peroxiredoxin domain and a C-terminal chitinase domain [19], which enables mucin binding and adhesion to the gut epithelial cells. CotE was mapped in silico for its CTL, HTL, and interferon- γ (IFN-γ) epitopes and used together with other CD proteins (SlpA, FliC) for designing a chimeric vaccine [20]. Bioinformatic methods confirmed its ability to induce the cytotoxic and helper T cells activation, along with the ability to induce IFN-γ, interleukin-2 (IL-2), as well as other proinflammatory cytokines (such as Tumor Necrosis Factor-α (TNF- α), IL-18, and IL-12) [20]. Other potential targets on the CD spore surface include: CdeC and CdeM (exosporium proteins) [21]; CotE, CotA, CotCB, CotL, and CDIF630_02480 (spore coat proteins) [16,22,23]. 

Another possible approach is to aim for proteins that are involved in the sporulation process. Bishop et al., showed that a genetic ablation in *clpP1/clpP2* genes coding caseinolytic protease P (ClpP) impaired the sporulation process [24]. SpoA is a key regulator of sporulation as its mutation leads to impaired CD growth and reduced spore production [25]. CspC is a soluble pseudoprotease that is used by the bacterium to sense bile acid germinants and other co-germinants, thus playing critical role in CD spore germination [26]. However, this approach still needs proper in vitro and in vivo validation to verify whether this protein is immunogenic and accessible for the antibodies. 

Thus far, none of the above vaccines have reached clinical trials that would indicate their effectiveness. It seems that targeting only spore components might not be enough to prevent the disease. A combined approach that includes toxins, spores, and some surface components of vegetative bacteria might likely be needed to combat the pathogen. Another complication in working with CD spores is their variability depending on the method of isolation and a lack of standardized methods for their characterization [27].

### 2.2. Vegetative CD Surface Proteins as Vaccine Candidates

Thus far, a lot of attention has been devoted to research on the immunoreactivity of CD surface proteins [10,28,29]. However, the search for new vaccine candidates is ongoing. There are multiple approaches of searching for new CD vaccine antigens: based on protein immunoreactivity with patients’ sera, based on its adhesive properties, a bioinformatic approach (reverse vaccinology), or a combined method. Some of these candidates have already been validated in the animal model of CDI. 

There are about 30 members of the Cell Wall Protein (CWP) family and they were shown to play a role in forming the S-layer but also in the interaction between CD and the host [30]. Cwp22 is a peptidoglycan cross-linking protein that takes part in adhesion and colonization. It is also essential for CD cytotoxicity and viability [31]. In our studies, we have shown that it is also immunoreactive and might serve as a suitable vaccine antigen. We identified three epitopes: ^54^EFRVAT^59^, ^201^KVNGKM^206^, and ^268^WQEKNGKKYY^277^, that were recognized by both CDI patients’ and umbilical cord blood sera [32]. These epitopes, after conjugation with a carrier, might be used in the new generation of subunit vaccines. Cwp66 is a cell wall protein that is the second major cell surface antigen, and its deletion affects the viability, motility, adhesion, virulence, and even antibiotic resistance of the bacterium [33]. It was previously shown that Cwp66 is an immunoreactive protein; moreover, specific anti-Cwp66 antibodies were identified in patients’ blood [28].

Another cell wall protein, CD0873, which was previously identified as a lipoprotein, was shown to act as an adhesin in in vitro studies [34]. Recently, Bradshaw et al., identified the CD0873 lipoprotein as a suitable vaccine candidate [35]. Treating mice with a CD0873-depleted CD strain resulted in impaired colonization rates when compared to the WT strain. Moreover, mice immunization with recombinant CD0873 induced high levels of specific IgG serum antibodies and IgA intestinal antibodies. As a result of intraperitoneal vaccination, mice did not develop diarrhea [35]. The same lipoprotein was displayed on liposomes (CD0873-MalLipo) and administered orally to hamsters. CD0873-MalLipo induced higher levels of neutralizing antibodies than unmodified lipoprotein and there was no detectable immunopathology in the hamsters’ gut [36]. 

Flagella is an important immunoreactive virulence factor in CD; however, it should be kept in mind that not all of the strains are flagellated. CD flagella is composed of FliC and FliD proteins that bind murine mucus, take part in biofilm formation, induce an innate immune response by TLR5 signaling, and are immunogenic in humans [37]. Because of their characteristics, they were already proposed as suitable vaccine antigens. A FliC-FliD fusion protein was shown to be protective in the mouse model of CDI [38]. Moreover, linear epitopes of FliC and FliD were mapped using a combined approach—bioinformatic prediction and mapping with human CDI sera [39]. Selected epitopes can be conjugated with a carrier protein and used in CD vaccines. This approach allows increasing vaccine safety by limiting the possibility of immunization with whole proteins that might induce unwanted side-effects such as cross- or autoimmune reactivity. 

PrkC is a membrane-bound serine/threonine kinase. Inactivation of the *prkC* gene resulted in changes in the morphology and the properties of the cell envelope, as well as increased sensitivity to antimicrobial compounds targeting the cell wall [40]. However, infection of hamsters with this Δ*prkC* strain resulted in only delayed colonization but did not affect the virulence. FeoB is another membrane-bound protein and an iron transporter that is needed for CD efficient toxin production (in vitro) and virulence (in vivo) [41]. Interestingly, the same protein was identified in the outermost exosporium-like layer of *C. difficile* 630 spores [23]. Thus far, there are no data on its immunoreactivity. Another recently discovered protein of dual localization is CD2831, which is a collagen-binding protein. Depending on the activity of specific ZmpI/PPEP-1 protease, it can be localized on the bacterial surface or released to the environment and possibly takes part in biofilm formation [42]. Moreover, it has a complement inhibiting activity making it even more interesting from the therapeutic point of view. Still, the data about its immunoreactivity should be collected. 

The development of bioinformatic techniques allowed the discovery of new candidates for peptide-based vaccines. It is worth mentioning that the bioinformatic approach is also useful in predicting possible cross-reactive or auto-reactive peptide sequences in a protein. First, the genome sequences are retrieved from databases such as NCBI (https://www.ncbi.nlm.nih.gov/, accessed on 20 November 2022), and tools such as VaxiJen (http://www.ddg-pharmfac.net/vaxijen/VaxiJen/VaxiJen.html, accessed on 3 January 2023) are used for testing the potential antigenicity of a protein. Then, proteins homological to human or microbiota can be eliminated from the list. In the next step, T-cell and B-cell epitopes are mapped using BCEpred (https://webs.iiitd.edu.in/raghava/bcepred/bcepred_submission.html, accessed on 20 November 2022) and IEDB (https://www.iedb.org/, accessed on 20 November 2022). Then, the immunogenicity is predicted and a set of analyses is performed such as molecular docking and 3-D structure prediction. With this approach, two new surface protein CD vaccine candidates were identified: subtilisin-like serine protease localized in the cell wall and flagellar hook associated protein FlgL [43]. However, there are no following data about the immunogenicity or immunoreactivity of these proteins. Another technique of reverse vaccinology was employed by Zhu et al., which resulted in the identification and characterization of a new surface vaccine target. Zhu et al., used Vaxign (https://violinet.org/vaxign/, accessed on 3 January 2023) to predict in silico suitable vaccine antigens based on their localization and adhesion properties. Based on the CD R20291 genome sequence, they found 31 candidates with the outstanding putative cell wall hydrolase (P_003217470.1, Cwl0971) [44]. Cwl0971 deletion mutant showed decreased bacteriolysis, toxin release, sporulation, and decreased fitness over the wild strain in the mouse infection model [44]. 

When using surface proteins as vaccine antigens, a question arises about the genetic variance among CD strains. It was previously shown, and it is confirmed by newest data, that the *slpA* gene [45] as well as *fliC* and *fliD* genes [46] are variable. 

## 3. Targeting Intracellular Proteins

Another group of potential vaccine antigens are heat-shock proteins. Both chaperones DnaK and GroEL from CD were shown to play a role in its virulence [47]. GroEL can be released or surface-associated, is immunogenic, and takes part in adhesion [48]. There are numerous examples of effective vaccines containing heat-shock proteins that elicit innate, humoral, and cell-mediated immunity [49]. However, on the other side, there is a high rate of sequence similarity between bacterial moonlighting proteins and the host’s own proteins, which might evoke unwanted autoimmune reactivity [50]. It was already suggested that these sequences should be analyzed in detail in terms of homology to other proteins or the epitopes should be mapped [51,52]. 

The M24 protein is an immunoreactive aminopeptidase recognized by CDI patients’ sera antibodies. Interestingly, its localization was predicted to be subcellular; however, other bacterial aminopeptidases were already detected on the bacterial surface acting as moonlighting proteins [53,54]. M24 was recently mapped for its B-cell epitopes. A peptide ^131^KKGIK^135^ conjugated to a carrier protein was shown to have immunogenic properties in vivo and was proposed to be a suitable vaccine antigen [55]. 

## 4. Targeting CD Surface Glycopolymers

CD surface glycopolymers define bacteria morphology, adhesion, and colonization. They are immunogenic and can generate an adaptive immune response [56]. They are also easily accessible on the surface and very often CD-specific [30]. Because of that, CD glycopolymers are considered to be promising vaccine antigens.

Much attention has been given to polysaccharide-based CD vaccine formulations in previous years [56]; however, there is still no such vaccine on the market. Cox et al., showed that lipoteichoic acid (LTA) and PS-II polysaccharides are highly conserved polymers of CD, visible on the bacterial surface and easily accessible to the immune system [57]. LTA alone was able to induce an immune response; however, PS-II needed a carrier protein for proper presentation. What is particularly interesting is that LTA and PS-II are both visible on the spore surface. 

A glycoconjugate CD vaccine based on synthetic oligosaccharides protected mice infected with two different CD strains [58]. Four synthetic antigens, ranging in size from disaccharides to hexasaccharides, were conjugated to CRM_197_, which is a carrier protein used in commercial vaccines. These vaccine candidates induced glycan-specific antibodies in mice and substantially limited CD colonization and colitis without disrupting the host’s microbiota. Passive transfer experiments with anti-PS-I serum revealed that protection is mediated by specific antiglycan antibodies; however, cell-mediated immunity likely also contributed to protection in vivo [58]. 

## 5. Using Non-Toxigenic Strains

NtCDMF is a membrane fraction of the nontoxigenic JND13-023 CD strain proposed to be a less costly alternative to purified antigens [59]. NtCDMF consists of many proteins, including SleB, which was able to induce high specific IgA titers following intrarectal administration. Senoh et al., showed on day 28 after immunization that there was 99% less CD in intrarectally vaccinated mice feces when compared to the control group. Moreover, hamsters immunized in the same way were partially protected from the lethal effects of CD toxins [59]. A NtTCD strain T7 was used as a vaccine platform to overexpress a colonization factor CD0873 and a domain of TcdB, whose spores were then administered to hamsters orally [60]. T7 with overexpressed CD0873 colonization factor effectively induced specific intestinal antibodies, which lowered the adhesion of pathogenic CD to Caco-2 cells. Another nontoxigenic CD strain named Z31 was used in piglets by Oliveira et al. [61]. CD Z31, which was originally isolated from a healthy dog, was orally administered to piglets and effectively reduced CDI clinical signs and the occurrence of mesocolonic edema in piglets. Moreover, no toxins were detected after challenge in the Z31-treated group [61]. The authors suggest competitive exclusion as the main mechanism of Z31 action. However, no humoral parameters were evaluated in this study, so it is impossible to assess if there were also specific protective antibodies produced. It is worth mentioning that there is a NtTCD strain M3 that successfully passed the phase II clinical trial (NCT01259726) in which it was tested as a prevention against recurrent CDI. 

## 6. Conclusions

The vaccine market is currently dominated by pediatric vaccines, but the dynamically changing demographic structure of the society, in favor of the elderly, causes a great need for the development of vaccines intended for this group of patients. Thus far, none of the novel CD vaccine antigen approaches characterized here have been assessed in clinical studies. However, the doubtful success of toxin-based vaccines shows that additional targets should be used to obtain an effective vaccine. Moreover, this new generation of antigens has many advantages (Table 1). One of the questions is which approach is most suitable and will be resistant to rapid genomic changes of *C. difficile*. More research is needed in order to deal with their limitations. Another possible future trend would be the use of CD extracellular vesicles (EVs) as a possible mucosal anti-CD vaccine. However, the biological characterization of EVs needs to be performed. 

## Figures and Tables

**Table 1:** Advantages and disadvantages of different types of CD vaccine antigens.

Vaccine Antigen Type	Advantages	Disadvantages
**Surface protein of the spore**	Possible destruction of spores prior to germination [18]; no need to use whole inactivated spores.	Limited efficacy of vaccination when used alone [62]; spore coat proteins masking surface antigens [16]; lack of knowledge on the interaction between spores and immune system.
**Surface protein of the vegetative cell**	No need to use whole inactivated bacteria; proteins accessible for antibodies; potent immune stimulators.	Variability in surface proteins among strains (SLPs), also due to homologous recombination [63].
**Intracellular proteins**	Major immunogens, highly expressed during infection.	High amino acid sequence homology to similar proteins in other organisms; possible autoimmune responses [52,64]; epitope mapping might be needed [55].
**Surface glycopolymers**	Immunogenic, conserved structures, easily accessible on the surface [57]; possible chemical synthesis.	Typically, a carrier for proper presentation is needed.
**Non-toxigenic strains**	Administration well-tolerated; strains compete for the same niche; stimulates mucosal responses [65].	Acquisition of toxin genes is possible by horizontal gene transfer [66].

## Data Availability

Not applicable.
